# Influence of non-ventilating intervals during early incubation stage on egg hatching process

**DOI:** 10.14202/vetworld.2023.1534-1540

**Published:** 2023-07-31

**Authors:** Wesam A. Fares, Mona R. M. Ahmed, R. E. Rizk, E. H. A. Shahein, N. G. Boutrous, Karim El-Sabrout

**Affiliations:** 1 Department of Poultry Breeding, Animal Production Research Institute, Agriculture Research Center, Giza, Egypt; 2 Department of Poultry Production, Faculty of Agriculture, Alexandria University, Alexandria, Egypt

**Keywords:** albumen pH, carbon dioxide, embryonic hormones, hatchability, non-ventilation

## Abstract

**Background and Aim:**

The exchange of oxygen and carbon dioxide (CO_2_) in the incubator plays a key role in embryonic development and hatching. This study aimed to study the effect of non-ventilated (NV) intervals during the early stage of embryonic development on the hatching process.

**Materials and Methods:**

Hatching eggs (n = 7200) were equally divided into four treatment groups and incubated in four incubators. The first group was incubated in normal ventilated condition (V) during the setting phase of incubation. Ventilation holes of the three remaining incubators were closed for the first 3, 6, and 9 days and termed as NV groups (NV1, NV2, and NV3, respectively). A gradual increase in CO_2_ was allowed for NV groups, followed by opening the incubator holes to permit ventilation throughout the rest of the incubation periods.

**Results:**

Obtained results demonstrated that CO_2_ concentration gradually increased up to 0.19% for the NV1 group, 0.41% for the NV2 group, and 0.90% for the NV3 group, while CO_2_ concentration remained at 0.08% during the first 9 days of incubation in the V group. Albumen pH was lowered for all NV groups. The highest hatchability percentage was recorded for NV3 followed by NV2 and NV1 groups. All NV groups represented earlier and narrower spread of hatch and higher hatched chick weight. Embryos and hatched chicks in the NV groups had higher hormonal levels of thyroxin and corticosterone.

**Conclusion:**

All non-ventilation periods had positive effects on narrowing the spread of hatch, increasing hatched chick weight and hatchability percentage compared to the normal V condition. Furthermore, the non-ventilation throughout the first 9 days of incubation yielded the best hatching results.

## Introduction

Investigating environmental conditions and their effects on the bird’s productive performance and welfare is very important for improving and advancing the global poultry industry [1, 2]. Therefore, the poultry industry’s main goals are to improve the efficiency of incubation processes and the quality of day-old chicks [3, 4]. Embryonic development is a process determined by genetic background and environmental conditions. Normally, the incubation conditions of oxygen (O_2_) and carbon dioxide (CO_2_) are 21% and 0.5%, respectively [5]. Carbon dioxide is an important gas in embryonic development which is released first during incubation from egg albumen as a natural reservoir and as the metabolic by-product of the embryos [6, 7]. In addition, Sadler *et al*. [8] found that 4% CO_2_ could enhance embryonic growth during the first 48 h of incubation and improve amnion closure during early incubation. Furthermore, Reijrink *et al*. [9] found that hypercapnia conditions through the first 5 days of incubation decreased albumen pH and egg weight loss.

Concerning incubation conditions, previous studies have shown that non-ventilation in the first 10 days of incubation allowed a gradual increase in CO_2_ up to 1.5%, and consequently reduced albumen pH, as well as enhanced embryonic growth and improved hatchability [10, 11]. Moreover, raising CO_2_ levels during the first part of incubation influences albumen acidification and liquefaction along with sub-embryonic fluid formation [12]. It decreases the barrier to the diffusion of O_2_ to the embryo [13]. Working on duck eggs, El-Hanoun *et al*. [14] concluded that non-ventilation conditions with a circulation of CO_2_ for the first 10 days of incubation are preferable to ventilation conditions for hatchability results. Prado-Rebolledo *et al*. [15] mentioned that hatchability percentage and broiler chick size significantly increased with 3000 ppm CO_2_ concentration. Mortola [16] showed that short-term hypercapnia could influence lung function causing an early pipping. In addition, Fares *et al*. [17] and Liu *et al*. [18] reported that the gradual increase of CO_2_ during the first 10 days of incubation caused higher hormonal levels of triiodothyronine (T_3_), tetraiodothyronine (T_4_), and corticosterone and these hormones could have a cardinal role in improving hatchability and narrowing hatch time.

This study aimed to determine the possible beneficial effect of non-ventilation conditions for 3, 6, and 9 days on embryonic mortality, spread of hatch, hatch time, hormonal levels, and hatchability in Mandarah breeder chickens.

## Materials and Methods

### Ethical approval

All procedures and husbandry guidelines were performed according to the Experimental Animal Care Committee Ethics of Animal Production Research Institute and Alexandria University (AU-092304303).

### Study period and location

This study was conducted from September 2022 to October 2022 at El-Sabahia Poultry Research Station, Animal Production Research Institute, Agricultural Research Center, Egypt.

### Experimental design

Hatching eggs (n = 7200) produced from Mandarah breeder chickens (an Egyptian-developed dual-purpose strain), aged 45 weeks were collected and stored for 5 days. Eggs were weighed and randomly/equally divided into four incubators, whereas 1800 hatched eggs were divided into six replicates for each incubator. The first egg group was incubated in a normally ventilated (V) incubator (V) during the setting phase (0–18 days) and served as a control group. Ventilation holes of the incubators were closed for 3, 6, and 9 days for the rest of the treatment groups and termed as non-ventilated (NV) groups (NV1, NV2, and NV3, respectively). All eggs were incubated at 37.5°C and 55% relative humidity during the setting phase. Gradual increase of CO_2_ was allowed among the experimental treatment groups, followed by opening the incubator holes to permit ventilation until the end of the incubation periods. Carbon dioxide concentration in the incubators was measured per hour using a CO_2_ sensor (CO_2_ Meter-EZTCH-GCH, 2018, USA). Setting eggs’ time in the incubator was determined to obtain hatch time in an hour and was considered as 0 h. At 432 h of incubation, the eggs were weighed and candled for transferring the living embryos to the hatcher.

### Measurement of albumen pH

The albumen pH was measured by pH meter for five fresh eggs per replicate among all experimental groups before setting and considered as 0 time. Albumen pH values were measured in eggs of V and NV1 at the end of the 3^rd^ day of the incubation, eggs of V, NV1, and NV2 at the end of the 6^th^ day, and eggs of V, NV1, NV2, and NV3 at the end of the 9^th^ day of the incubation. The germinal disc of fertile eggs was identified using a magnifying lens.

### Egg weight

Eggs were individually weighed (g) on 0, 9, and 18 days of incubation, and the percentages of egg weight loss among incubated intervals (0–9, 10–18, and 0–18 days) per each incubator were calculated.

### Hatching events

The hatcher was opened at 456 h and repeated every 4 h. Hatched chicks were wing banded, weighed, and recorded as chick weight at hatch, and then placed back in the incubator after recording the hatching time. The hatch window was monitored as the time elapsed between the first and last chicks, and it included the average hatch time (h) and hatch time range (h). The range of hatch time was calculated as the difference between maximum and minimum hatch times. Moreover, between 456 and 521 h as hatch time, hatching eggs were individually checked every 4 h to determine the spread of hatch and calculated as the percentage of hatched chicks through four periods and termed as early, peak, late, and post-late hatched chicks. The chicks were left in the incubator until servicing time (termination of incubation) and weighed again at the time of removal from the hatcher and termed as chick weight at pull-out. Chick body weight loss percentage was calculated according to the following equation:

Chick weight at hatch - Chick weight at pull-out/Chick weight at hatch × 100.

The hatchability of fertile egg percentage was also recorded according to the following equation:

Number of chicks/Number of fertile eggs × 100.

### Embryonic mortality

Eggs that failed to hatch were broken out and examined macroscopically to estimate the embryonic age and assigned according to death time in days as possible. The percentage of embryonic mortality expressed as a percentage of fertile eggs was recorded and classified for periods (1–5, 6–10, 11–15, and 16–21 days) and at pipping.

### Hormonal levels

Blood samples were collected from three embryos and three hatched chicks per each replicate on the 16 and 18^th^ days of embryonic age, at pipping and hatching. The samples were centrifuged, and the collected serum was stored at −20°C until the detection of triiodothyronine (T_3_), tetraiodothyronine (T_4_), and corticosterone hormone levels. Serum T_3_ and T_4_ (ng/mL) were determined by radioimmunoassay according to the method of Darras *et al*. [19]. Corticosterone concentration (ng/mL) was determined according to Weimer *et al*. [20].

### Statistical analysis

Data were statistically analyzed using a one-way analysis of variance implemented in SAS version 15.1 (SAS Institute Inc., Cary, North Carolina, USA) [21] using the general linear model procedure. Mean differences were tested by Tukey’s test for comparison at p ≤ 0.05 significant level.

The following model was used:

Y_ij_ = μ + H_i_ + e_ij_

Y_ij_ = observed traits, µ = the overall mean, H_i_ = the effect of ventilation condition, and e_ij_ = random error.

## Results

### Carbon dioxide concentration

**Figure-1:**
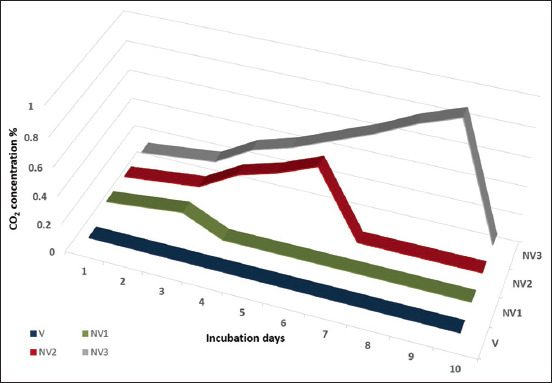
Carbon dioxide concentration in ventilated and non-ventilated incubators through the 10 days of incubation.

Figure-1 depicts the concentration of CO_2_ as measured in continuous days for V and NV incubators through the first 10 incubation days. This figure also reveals that CO_2_ concentration gradually increased from 0.1 on the 1^st^ day to 0.19 on the 3^rd^ day of incubation for the NV1 group. It gradually increased up to 0.41% on the 6^th^ day of incubation in the second experimental group (NV2). Moreover, the third group (NV3) represented a greater rise in CO_2_ level from 0.1 to 0.9 on the 9^th^ day of incubation. While in the V group, CO_2_ concentration remained at about 0.08% constant and low through the first 9 days. After opening the holes for all NV incubators, CO_2_ concentration returned to the normal condition as those in the V one.

### Albumen pH

**Figure-2:**
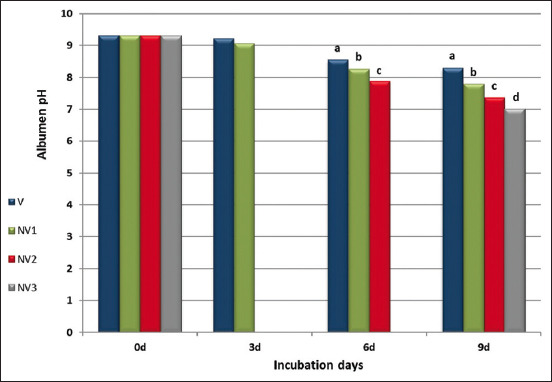
Egg albumen pH in ventilated and non-ventilated incubators through the end of incubated days. ^a,b,c,d^Means having different letters in the same row are significantly different (p ≤ 0.05).

Figure-2 illustrates albumen pH values for V and NV incubators at the end of incubated days. All experimental egg groups represented the same response of albumen pH (9.30) for eggs before setting. At the end of the 3^rd^ day of incubation, albumen pH values numerically decreased for the NV1 group compared with the V group. Concomitantly with the increasing level of CO_2_ on the 6^th^ day of incubation, albumen pH significantly (p ≤ 0.05) decreased for the NV2 group followed by NV1 compared to the V group. Furthermore, on the 9^th^ day of incubation, the increase in CO_2_ in the NV3 incubator significantly (p ≤ 0.05) decreased albumen pH compared to NV1, NV2, and V groups.

### Egg weight loss and embryonic mortality

The effects of non-ventilation periods on egg weight loss percentages and embryonic mortality of incubated eggs are shown in Table-1. It appears that the percentage of egg weight loss was significantly (p ≤ 0.05) decreased among incubation intervals (0–9 and 0–18 days) for all NV experimented times compared with those for the V group. During 10–18 incubation days, egg weight loss percentages were (p ≤ 0.05) decreased for eggs exposed to NV2 and NV3 conditions compared to the V group. Eggs subjected to the NV3 condition had the lowest egg weight loss (%), followed by NV2 and NV1.

**Table-1:** Effect of non-ventilation periods in the incubators on egg weight loss and embryonic mortality.

Interval periods	NV			V	p-value
	
	0–3 d (NV1)	0–6 d (NV2)	0–9 d (NV3)	0–18 d	
Egg weight loss (%)					
0–9 (d)	7.54^b^ ± 0.70	6.95^c^ ± 0.07	6.00^d^ ± 0. 06	8.04^a^ ± 0.08	<0.000
10–18 (d)	6.17^ab^ ± 0.15	5.89^b^ ± 0.05	5.01^c^ ± 0.07	6.46^a^ ± 0.07	<0.000
0–18 (d)	13.75^b^ ± 0.07	12.91^c^ ± 0.08	11.05^d^ ± 0.06	14.54^a^ ± 0.06	<0.000
Embryonic mortality (%)					
1–5 (d)	4.49^b^ ± 0.32	4.14^b^ ± 0.04	3.93^b^ ± 0.06	7.60^a^ ± 0.05	<0.000
6–10 (d)	2.17 ± 0.58	2.06 ± 0.11	1.20 ± 0.28	2.79 ± 0.63	0.062
11–15 (d)	0.50^b^ ± 0.05	0.00^c^ ± 0.00	0.00^c^ ± 0.00	2.04^a^ ± 0.08	<0.000
16–21 (d)	2.99^b^ ± 0.06	2.99^b^ ± 0.01	2.83^b^ ± 0.08	4.81^a^ ± 0.58	<0.007
Pipped eggs	1.70^b^ ± 0.08	1.08^c^ ± 0.09	0.39^d^ ± 0.01	3.00^a^ ± 0.05	<0.000

^a,b,c,d^Means having different letters in the same row are significantly different (p ≤ 0.05), d=Day, NV=Non-ventilated, V=Ventilated

Throughout the experimental periods (1–5, 11–15, 16–20, and pipped eggs), the incubator’s ventilation during the setting phase (0–18 days) significantly (p ≤ 0.05) increased embryonic mortality compared with the NV groups.

### Hatching output

Table-2 shows data related to hatching output as affected by non-ventilation periods in the incubators. The non-ventilation period during the first 9 days of incubation represented the highest (p ≤ 0.05) percentage of hatchability for fertile eggs (92.03%), followed by 90.05% for NV2 and 89.15% for NV1 groups compared to 80.18% for the V group. Regarding the spread of hatch, hatched chick percentages at mid-hatch were significantly (p ≤ 0.05) higher for all non-ventilation periods compared to those for the V condition, and the ranking order for hatchability percentages was recorded for NV3, NV2, and NV1, respectively. However, the V group had higher (p ≤ 0.05) hatched chick percentages for late and post-late times than the NV groups. Referring to the hatch window, the average hatch time was significantly (p ≤ 0.05) decreased by 7.5 h for NV1, 12.02 h for NV2, and 17.95 h for NV3 compared to the V group. Furthermore, the range of hatch time was significantly (p ≤ 0.05) shortened by 4.49 h for NV1, 6.27 h for NV2, and 7.92 h for NV3 compared to the V group. Hatched chicks produced under NV conditions had the largest body weights at hatch compared with the V group. Furthermore, non-ventilation conditions increased chick body weights at pull-out, and the NV3 group had the highest (p ≤ 0.05) record. Chick weight loss was significantly (p ≤ 0.05) higher in NV1 and V groups compared to NV2 and NV3 groups, while the NV3 group had the lowest chick weight loss.

**Table-2:** Effect of non-ventilation periods in the incubators on hatchability, spread of hatch, hatch window, and hatched chick weight.

Traits	NV			V	p-value
	
	0–3 d (NV1)	0–6 d (NV2)	0–9 d (NV3)	0–18 d	
Hatchability of fertile eggs %	89.15^a^ ± 1.37	90.05^a^ ± 1.09	92.03^a^ ± 0.73	80.18^b^ ± 1.46	0.014
Spread of hatch^1^					
Early hatch %	18.27 ± 0.23	19.01 ± 0.43	18.05 ± 0.05	17.99 ± 0.29	0.215
Middle hatch%	72.43^c^ ± 0.36	74.00^b^ ± 0.32	77.61^a^ ± 0.22	47.00^d^ ± 0.47	0.001
Late hatch%	5.97^b^ ± 0.35	4.99^b^ ± 0.35	2.48^c^ ± 0.28	21.99^a^ ± 0.28	0.001
Post late hatch%	3.32^b^ ± 0.20	1.99^c^ ± 0.35	0.99^d^ ± 0.01	13.0^a^ ± 0.38	0.001
Hatch window (h)					
Average hatch time	489.29^b^ ± 1.27	484.77^b^ ± 2.28	478.84^c^ ± 2.37	496.79^a^ ± 1.46	0.001
Range of hatch time^2^	42.85^b^ ± 1.59	41.07^b^ ± 3.18	39.42^b^ ± 1.82	47.34^a^ ± 0.73	0.001
Hatched chick weight (g)					
Chick weight at hatch	38.26^b^ ± 0.97	39.70^ab^ ± 0.42	40.09^a^ ± 0.94	36.89^b^ ± 0.47	0.002
Chick weight at pull-out	36.18^a^ ± 0.97	37.95^a^ ± 1.08	38.90^a^ ± 1.23	34.75^b^ ± 0.34	0.001
Chick weight loss %	5.43^a^ ± 1.30	4.40^b^ ± 1.29	2.96^c^ ± 1.40	5.80^a^ ± 0.06	0.001

^a,b,c^Means having different letters in the same row are significantly different (p ≤ 0.05), 1-Spread of hatch: V (early: 473.12–485.12 h, peak: 485.12–97.12 h, late: 497.12–509.12 h and post-late: hatch: 509.12–520.46 h); NV1 (early: 467.87–479.87 h, peak: 479.87–491.87 h, late: 491.87–503.87 h, and post-late hatch: 503.87–510.72 h); NV2 (early: 464.24–476.24 h, peak: 476.24–488.24 h, late: 488.24–500.24 h, and post-late hatch: 500.24–505.31 h), and NV3 (early: 459.13–471.13 h, peak: 471.13–483.13, late: 483.13–495.13 h, and post-late hatch: 495.13–498.55 h). 2-Range of hatch time=maximum hatch time - minimum hatch time. d=Day. NV=Non-ventilated, V=Ventilated

### Hormonal levels

Table-3 shows the effect of non-ventilation intervals during egg incubation on T_3_, T_4_, and corticosterone hormones for embryos and hatched chicks. The T_3_, T_4_, and corticosterone hormones increased (p ≤ 0.05) in embryos aged 17 and 18 days. They also increased in pipped and hatched chicks of non-ventilation groups compared with the V group, and the NV3 group showed the highest levels of the studied hormones.

**Table-3:** Effect of non-ventilation periods in the incubators on triiodothyronine, thyroid, and corticosterone hormones for embryos and hatched chicks.

Traits	NV			V	p-value
	
	0–3 d (NV1)	0–6 d (NV2)	0–9 d (NV3)	0–18 d	
Triiodothyronine (T_3_, ng/mL)					
Embryos at 17^th^ d	2.62^b^ ± 0.05	2.70^b^ ± 0.04	2.90^a^ ± 0.04	2.12^c^ ± 0.03	<0.000
Embryos at 18^th^ d	2.81^c^ ± 0.11	3.05^b^ ± 0.02	3.65^a^ ± 0.02	2.30^d^ ± 0.04	<0.000
Pipped chicks	3.35^b^ ± 0.02	3.50^b^ ± 0.08	3.85^a^ ± 0.02	2.70^c^ ± 0.04	<0.000
Hatched chicks	2.40^c^ ± 0.04	2.55^b^ ± 0.02	2.85^a^ ± 0.02	2.05 ^d^ ± 0.02	<0.000
Thyroid (T_4_, ng/mL)					
Embryos at 17^th^ d	4.75^c^ ± 0.06	5.00^b^ ± 0.04	5.75^a^ ± 0.02	3.45^d^ ± 0.02	<0.000
Embryos at 18^th^ d	7.65^b^ ± 0.02	7.90^b^ ± 0.08	8.40^a^ ± 0.08	4.15^c^ ± 0.15	<0.000
Pipped chicks	8.50^c^ ± 0.09	8.90^b^ ± 0.13	9.85^a^ ± 0.02	5.20^d^ ± 0.04	<0.000
Hatched chicks	4.50^c^ ± 0.04	4.85^b^ ± 0.11	5.80^a^ ± 0.08	3.30^d^ ± 0.04	<0.000
Corticosterone (ng/mL)					
Embryos at 17^th^ d	12.73^a^ ± 0.26	12.65^a^ ± 0.37	13.55^a^ ± 0.35	11.32^b^ ± 0.33	<0.000
Embryos at 18^th^ d	15.81^b^ ± 0.22	16.89^b^ ± 0.52	18.84^a^ ± 0.29	14.43^c^ ± 0.19	<0.000
Pipped chicks	18.58^b^ ± 0.44	20.22^b^ ± 0.72	22.78^a^ ± 0.62	16.41^c^ ± 0.20	<0.000
Hatched chicks	17.18^b^ ± 0.42	18.41^b^ ± 0.32	19.78^a^ ± 0.39	14.90^c^ ± 0.17	<0.000

^a,b,c,d^Means having different letters in the same row are significantly different (p ≤ 0.05), d=Day, NV=Non-ventilated, V=Ventilated

## Discussion

The non-ventilation procedure during the chicken egg incubation by closing ventilation holes for 3, 6, and 9 days resulted in a gradual increase in CO_2_ concentration (Figure-1), which corresponds to previous findings by different authors who closed ventilation holes at different times: For 2 days by Sadler *et al*. [8], 3 days by Õzlü *et al*. [22], 5 days by Reijrink *et al*. [9], and 10 days by Okur [11]. Increasing CO_2_ concentration during incubation could be due to a biological change in the CO_2_ curve caused by embryos’ area vasculosa, which begins gas exchange at an early stage of development, and then the chorioallantoic membrane develops and contacts with eggshell membranes, activating and starting the respiratory function as demonstrated by Tona *et al*. [23] and De Smit *et al*. [24]. The most notable observation of this study was the remarkable embryonic tolerance to increasing CO_2_ concentrations during the early stage of embryogenesis and this notion was approved and explained by Taylor and Kreutziger [25], who revealed that this tolerance action relies on augmented CO_2_ tension that would quicken calcium mobilization and in turn increase bicarbonate buffering capacity.

Supporting our findings regarding the decrease in albumin pH due to non-ventilation conditions is illustrated in Figure-2. Reijrink *et al*. [9] and Bruggeman *et al*. [12] have drawn the same reported conclusion. Moreover, forming H^+^ protons and bicarbonate ions due to the chemical reaction of H_2_O with ambient CO_2_ in albumen is the main reason for albumen acidification and decrease in albumen pH, as explained by Bruggeman *et al*. [12].

In general, the obtained results of the decrease in egg loss during incubation by non-ventilation conditions (Table-1) are in agreement with earlier studies regarding closing the incubators for different intervals representing hypercapnia conditions, as for 10 days [17, 24], for 5 days [9], and for 3 days [22]. The decrease in egg loss could be due to CO_2_ accumulation in the incubator which creates a masking layer of CO_2_ on the eggshell surface and impeded the water eggshell conductance and then affected egg weight loss for NV eggs as stated by Fares *et al*. [17]. Furthermore, the results of embryonic mortality reduction for NV groups versus those of the V group are consistent with those previously mentioned by El-Hanoun *et al*. [14] and Herrera *et al*. [26]. The embryonic death reduction could be due to the albumen pH reduction, as represented in Figure-1, and related to the albumen quality due to NV condition and this conclusion was previously documented by Romanoff [27]. In addition, the increase in embryonic mortality for the V group could be related to the egg loss increase for this group and this notion added credence to the study of Rizk *et al*. [28], who stated that the egg weight loss during incubation might be related to embryonic mortality. By matching the results of Figure-1, Tables-2 and 3, it is possible to conclude that the increase in CO_2_ in the incubator during non-ventilation conditions is the reason of the increase in T_3_, T_4,_ and corticosterone hormones, which gain additional benefits such as hatchability improvement, hatch time confining, and narrowing hatch time spread. The significant improvement of hatchability percentages in NV groups, as shown in Table-2, can be explained in the light of embryonic mortality reduction, as shown in Table-1, and albumen pH reduction in NV groups (Figure-1), which was caused by the hypercapnia condition previously documented by Fares *et al*. [17] and Decuypere *et al*. [29]. Similar to our findings, Okur *et al*. [30] indicated that increasing CO_2_ concentrations during the first 10 days of incubation resulted in an improved hatchability percentage. In addition, De Smit *et al*. [24] and Tona *et al*. [31] revealed that the condition of NV at the beginning of the incubation might be the reason for the higher partial pressure of O_2_ and CO_2_ in the air cell which led to hatchability improvement. Furthermore, this improvement could be due to the CO_2_ retarded thick albumen layer and apparent breakdown of the chalaza membrane as mentioned by Sadler *et al*. [8]. Burley and Vadehra [32] demonstrated that CO_2_ causes early liquefaction of albumen which facilitates the movement of nutrients to the embryos, as well as reduces any barrier of O_2_ diffusion to the embryos [13]. Different mechanisms of the physiological response of embryos and hatching improvement due to CO_2_ increase were detected by Everaert *et al*. [33] who mentioned that expression of a pH-dependent enzyme like carbonic anhydrase enzyme catalyzes the hydration of CO_2_ to bicarbonate and protons. Latter and Baggott [34] reported that the activity of carbonic anhydrase enzyme formed in the endodermal cells of area vascula supplies H^+^ that exchanges with Na^+^ which helps in the production of sub-embryonic fluids. In addition, Deeming [35] reported that sub-embryonic fluids play a pivotal role in embryo survival. Furthermore, hypercapnia may affect blood flow and the cardiovascular system [36, 37].

De Smit *et al*. [24] interpreted the relationship between increasing hormonal levels induced by non-ventilation conditions and improved hatching output, reporting that CO_2_ overflow is known to be a stimulus for higher T_3_ concentration, which leads to hatching initiation. Moreover, Ramachandran and McDaniel [38] found that accelerating pipping could be due to partial pressure increases of CO_2_ in the air cell. Decuypere *et al*. [39] mentioned that the interval between the internal pipping and hatching process is thyroxin dependence, whereas thyroid metabolism in embryos is stimulated by corticosteroids which affect the hatching process by hepatic 5-’D activity stimulation which helps the conversion of T_4_ to T_3_. In addition, Blacker *et al*. [40] stated that CO_2_ accelerates the development of the embryo along with early maturation of the surfactant system which controls lung surface tension leading to the first breath. In addition, lung surfactant system maturation is promoted by corticosterone hormone, besides phospholipid in the lung is markedly increased by T_3_ which stimulates the production of ornithokallikrien enzyme [39, 41]. The same authors also mentioned that increasing ornithokallikrien enzyme in the lung during the transition from allantoic to pulmonary respiration was a result of the higher partial pressure of CO_2_ in the air cell, and this enzyme increases the flow of lung blood which induces early internal and external pipping.

Higher chick weight at hatch and pull-out for NV groups compared to the V group could be explained by a decrease in egg weight loss for NV groups (Table-1). These results are consistent with the findings of Fares *et al*. [17], who demonstrated that hatched chick weight is affected by egg weight loss variations. Furthermore, Bilalissi *et al*. [42] concluded the same results of improving hatching weight due to the non-ventilation procedure. Likewise, hatch time may affect chick body weight at hatch, as previously mentioned by Bilalissi *et al*. [42] and Hopcroft *et al*. [43]. Therefore, shortening the hatch window range and hatch spread by the non-ventilation condition is favored to obtain good results of the hatch, as remaining the hatched chicks for a long period in the hatcher exposes the chicks to dehydration with losing a lot of water amount and increasing chick weight loss percentages as well as decreasing body weight at pull-out.

## Conclusion

Controlling CO_2_ in the incubator by closing the incubator holes for the first 3, 6, and 9 days of incubation is a good practical method for managing albumen pH, embryonic hormones, and hatchability percentage. Thus, non-ventilation through the first 9 days of incubation produced the best results compared with those for 3 or 6 days of non-ventilation and the ventilation conditions. This strategy of managing CO_2_ emission achieved the best results of hatch through decreasing egg and chick weight loss, narrowing the spread of hatch, diminishing embryonic mortality, and increasing hatchability percentage and hatched chick weight.

## Data Availability

The supplementary data can be available from the corresponding author upon a reasonable request.

## Authors’ Contributions

WAF and RER: Designed the study. WAF, MRMA, EHAS, and NGB: Conducted the animal trial and revised the manuscript. WAF and MRMA: Analyzed the samples. MRMA, KES, and NGB: Sample collection and data analysis. WAF, KES, and RER: Drafted the manuscript. EHAS and NGB: Revised the manuscript. All authors have read, reviewed, and approved the final manuscript.
